# Assessment of contrast perception of obstacles in a tunnel entrance

**DOI:** 10.15171/hpp.2018.38

**Published:** 2018-10-27

**Authors:** Ahmad Mehri, Somayeh Farhang Dehghan, Milad Abbasi, Mohammad Hosein Beheshti, Javad Sajedifar, Sayed Mohammad Jafari, Monireh Khadem, Roohalah Hajizadeh

**Affiliations:** ^1^Department of Occupational Health, Iranshahr University of Medical Sciences, Iranshahr, Iran; ^2^Department of Occupational Health, Shahid Beheshti University of Medical Sciences, Tehran, Iran; ^3^Research Center for Environmental Determinants of Health (RCEDH), Kermanshah University of Medical Sciences, Kermanshah, Iran; ^4^Students’ Scientific Research Center, Tehran University of Medical Sciences, Tehran, Iran; ^5^Department of Occupational Health, Gonabad University of Medical Sciences, Gonabad, Iran; ^6^Department of Occupational Health Engineering, Neyshabur University of Medical Sciences, Iran; ^7^Faculty of Medicine, Khatam Al-Nabieen University, Kabul, Afghanistan; ^8^Department of Occupational Health, Tehran University of Medical Sciences, Tehran, Iran; ^9^Occupational Health Research Center, Qom University of Medical Sciences, Qom, Iran

**Keywords:** Contrast perception, Safe stopping distance, Tunnel entrance, Ilam

## Abstract

**Background:** The purpose of this study was to assess the contrast perception of obstacles in a tunnel entrance which was placed in Ilam Province, Iran.

**Methods:** An obstacle with the reflection coefficient of 20% was mounted at the entrance of thetunnel and then, the car was placed near the tunnel entrance and the intrinsic luminance of the road (L_r,intrinsic_) and obstacle (L_o,intrinsic_) were measured inside the car using the luminance meter.

**Results:** Intrinsic luminance of obstacle and road at the entrance of the tunnel were measured as 41 and 17 cd/m^2^, respectively. The contrast perception of obstacle in deriver’s eyes and in the safe stopping distance (SSD) from the tunnel entrance was determined as 2.79 cd/m^2^. At the entrance of the studied tunnel, the contrast perception of obstacles with the reflection coefficient of 20% was lower than the minimum contrast perception (28%) recommended by International Commission on Illumination.

**Conclusion:** The main conclusion that can be drawn from it, is that this obstacle in the SSD from the tunnel entrance cannot be conceived by the drivers, which may lead to higher rate of road traffic crashes.

## Introduction

Road traffic accidents that occur in tunnels make up a small percentage of road accidents, but with enormous consequences.^1,2^ In the meantime, the accident rate in the entrance zone of tunnel is so high compared to other areas; such that the Norway Highway Bureau reported that about 63.7% of tunnel collisions occur at entrance zones.^2^ Many studies have suggested that the visual features for drivers approaching a tunnel entrance are crucial factors to assess the risk of crash related to tunnel travel.^1,3^ They indicated that difficulty in the visual adaptation of drivers caused by dark environment inside the tunnel is an important factor in this regard.^4,5^

Visibility is the key to ensuring traffic safety, since over 90% of the information needed while driving is obtained visually.^6^ According to studies, some environmental confounders that interfere with the visual function include the luminance of natural and artificial light sources. Luminance exceeding a certain level causes disability glare, leading to reduction in the driver’s visibility and can make drivers unable to see the objects ahead, so there could be an increased risk of traffic accidents.^7^ The drivers’ transient vision problems induced by the rapid change of illumination at a tunnel’s entrance has been reported by Du et al.^8^

Requirement of tunnel entrance lighting has attracted wide interest. The standards for tunnel lighting vary across countries; however, International Commission on Illumination Report CIE 088-2004 is the most agreeable and acceptable one among them.^9^ In a study on the tunnel lighting settings conducted by Pachamanov and Pachamanova, it was shown that the lighting installations and illumination transitions are curve fitting that meets the requirements of brightness and glare.^10^

In general,glare can be divided into 2 types, disability glare and discomfort glare. Disability glare is due to scattered light into the eye and its expansion as a large veil on retina reduces the contrast of images, and therefore the visual quality. In spite of that, the discomfort glare is uncomfortable but does not disrupt visibility and interfere with the visual function.^11,12^

Disability glare at the entrance of road tunnel is due to the equivalent veiling luminance (nature surrounding the tunnel and artificial structures), car windshield luminance, and atmospheric luminance (Figure S1; see Supplementary file 1). It can lead to decrease in the contrast of obstacles and lack of recognition by the drivers.^13^ Some research studies showed that the presence of glare sources may have detrimental effect on driver’s behavior, such as reducing the speed of vehicle and the inability to control it.^14,15^ A study by Ranney et al indicated that the glare causes reduction in the visibility of drivers to recognize the pedestrians on the road.^16^

Perceived contrast is created in the retina because of luminance difference in perception of the road and obstacle.^17^ Drivers must be able to see obstacles with the reflection coefficient of 20% in the safe stopping distance (SSD) from the tunnel entrance. In other words, the minimum perceived contrast into the driver’s eyes is 28% to see the obstacles easily. According to CIE-088-2004 standard,^9^ some environmental confounding factors such as equivalent veiling luminance, car windshield luminance, and atmospheric luminance reduce the perceived contrast of the obstacle, resulting in the disability glare and non-visibility of that obstacle.^18^

The aim of this study was to assess the contrast perception of obstacles in a tunnel entrance which was placed in Ilam, Iran and also assess the effects of luminance (equivalent veiling luminance, car windshield luminance and atmospheric luminance) on perceived contrast of the obstacle in the tunnel entrance. The characteristics of studied tunnel have been described in the study of Mehri et al.^1^

## Materials and Methods

### Making an obstacle with reflection coefficient of 20%

The first step in evaluating the perceived contrast of obstacle at the tunnels entrance is the preparation of an obstacle with reflection coefficient of 20%.^18^ In dark environment of the laboratory (3×4×3 m; without window), the cardboards (20×20 cm), as the obstacles, with different colors were exposed to radiation of LED light source, with color temperature of 6400 K and Color Rendering Index (CRI) of 80%. Then, the lighting of obstacle (E) and reflective luminance (L) of obstacles were measured by a Lux meter (HAGNER, model EC1; Sweden) and Luminance meter [HAGNER, Model S3; Sweden], respectively. Then, the reflection coefficients [ρ] of different obstacles were calculated using equation 1.^18^

(Eq. 1) $$\rho =\frac{L.\text{​}\pi }{Ε}$$

The obstacle which had the reflection coefficient of 20% was selected as the test obstacle. Its lighting characteristics are presented below:

Illuminance: 255 Lux

Luminance: 16 cd/m^2^

Reflection coefficient: 20%

### Safe stopping distance

The scientific definition of the SSD is the sum of distance between processing the obstacle in the brain and the appropriate reaction of driver to brake and stop the car before hitting the obstacle.^9,19^ Safe stopping distance depends on several factors, including road gradient (upward or downward; ± *s*, %), passing vehicle speed limit (*U*, m/s), driver reaction time (*t*0, s), acceleration due to gravity (*g*, m/s^2^), and the coefficient of friction between the tire and the road (*f*, dimensionless).^1^ Eq. 2 is used to calculate the SSD.

(Eq. 2) $$SSD=\;U.t_{0\;}\;+\;\frac{U^{2}}{2.g.\left[f\pm s\right]}$$

The coefficient of friction between the tire and the road that depends on the vehicle speed and the road surface [dry or wet], is estimated as shown in Figure S2 (see Supplementary file 1).^9^ The mean reaction time for drivers was considered as one second, according to CIE (Commission Internationale de l’Éclairage) standard.^9,20^ Then, based on the recommendation of Parise et al for estimating the coefficient of friction between the road and the tire, road surface can be considered wet where the average annual rainfall is over 75 hours.^21^ Due to the location of the tunnel in the city of Ilam and according to the 10-year information obtained from meteorological station 40 780 in Ilam, the average annual rainfall is 73.8 days. Other required variables to determine the SSD, include:

Maximum speed limit of vehicles: 18 m/s

Road gradient (upward): -0.03

Reaction time: 1 s

Friction coefficient tire-road: 0.35

According to the above data, SSD was obtained as 69.6 m.

### Equivalent veiling luminance (L_seq_)

Equivalent veiling luminance (from the nature surrounding the tunnel) into the retina leads to decrease in the contrast of internal obstacles in the tunnel. In this study, the equivalent veiling luminance was estimated by a camera (model 108, 35 mm lens reflex; YASHICA) which meets the criteria of the CIE-088 standard. This camera can take pictures of the tunnel entrance at approximately 56° horizontally and 38° vertically in the surroundings of the tunnel.

The camera was mounted in a SSD from the tunnel entrance and at a height of driver’s eyes for taking photo of the entrance of the tunnel. The eye height of Iranian drivers was determined to be 131 cm, and the measurement was taken from the seat height of the conventional vehicles in Iran.^1,5^

**Figure 1:**
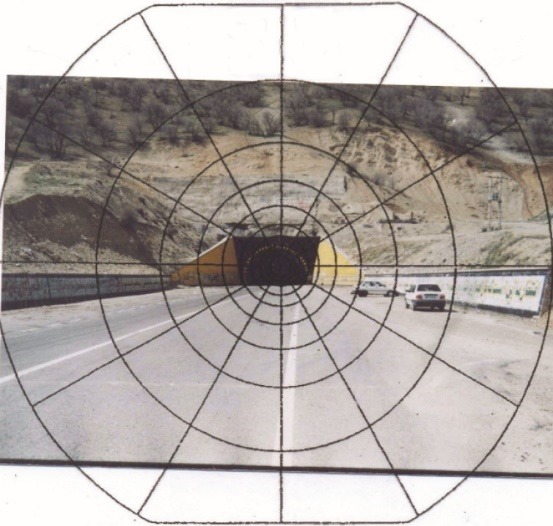
The Holladay polar diagram drawn on the photo of tunnel entrance.^1^

Next, the Holladay polar diagram (Figure 1) was drawn on the photo of the tunnel entrance, each sector of the rings was reticulated, and the percentage of environmental factors (rock, grass, road, etc) were determined in each sector. Then, considering the geographical direction of vehicles in the northern hemisphere and environment surrounding the tunnel, the luminance of each sector was estimated via the CIE-088 standard. The luminance of each ring was determined by adding the luminance of all its sectors.^9^ After determining the luminance of all rings (∑L_ije_), the equivalent veiling luminance (L_seq_) was calculated by equation 3. It is noteworthy that the Holladay polar diagram has 9 rings and each ring is divided into 12 sectors. The angle of each ring is shown in Table 1.^22^

L_seq_=5.1×10^-4^∑L_ije_ (Eq. 3)

### Atmospheric veiling luminance

**Figure 2:**
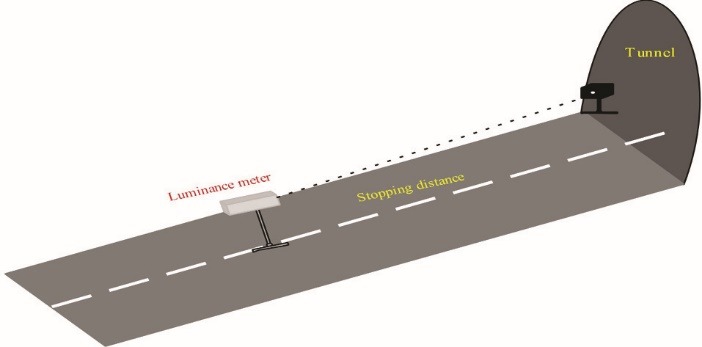
Determination of the atmospheric veiling luminance around the tunnel.^23^

Atmospheric veiling luminance is the luminance resulting from factors such as dust in the atmosphere that causes light scattering. According to the CIE, a black chamber with a small hole was used to determine the atmospheric veiling luminance (L_atm_) on a summer noon around the tunnel (Figure 2). At the SSD from the chamber, the hole was targeted by a luminance meter (HAGNER, Model S3; Sweden) and the luminance of confounding factors in the atmosphere was measured.^21^

### Windshield veiling luminance 

**Figure 3:**
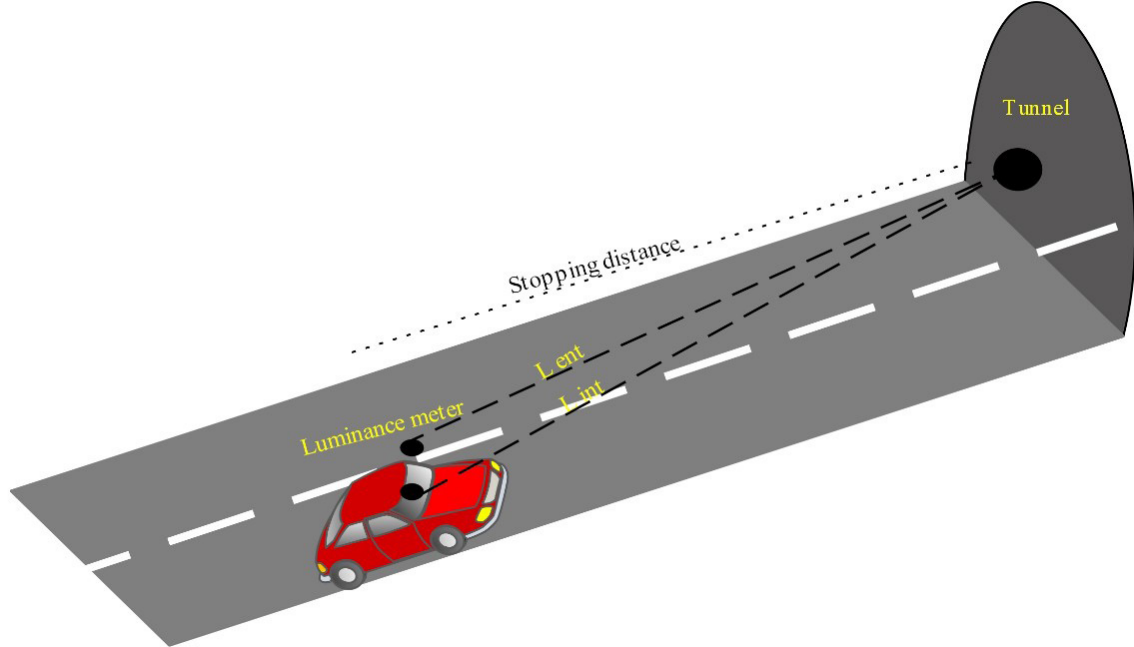
Determination of windshield veiling luminance near the tunnel entrance.^23^

One of the main factors reducing obstacles contrast into the driver’s eyes is the light scattering of vehicles windshield. In this study, to measure the windshield veiling luminance (L_ws_) on summer noon, the vehicle was placed at the entrance of the tunnel and in the SSD. Then, a fixed dark point on the inner wall of the tunnel was selected to measure the luminance in the outside (L_ext_) and inside (L_int_) of the vehicle (Figure 3). Thereafter, the windshield veiling luminance was determined using equation 4. It is notable that, according to different studies and CIE-088-2004 standard, windshield transmission coefficient (τ_ws_) was considered as 0.8.^9,21^

*L*_ws_ *= L*_in_ *- (π*_ws_ *× L*_ext_ *) * (Eq. 4)

### Intrinsic luminance of road and obstacle

**Figure 4:**
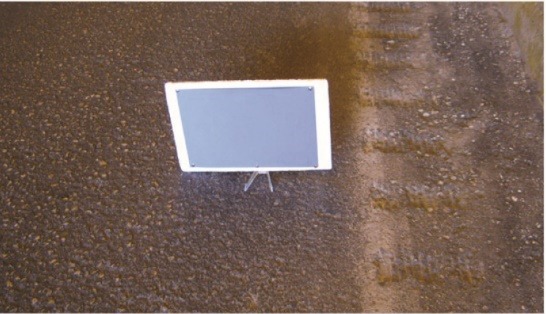
An obstacle with the reflection coefficient of 20%.^23^

Intrinsic luminance of the road (L_r,intrinsic_) and obstacle (L_o,intrinsic_) include the actual measurement of luminance of the road surface and obstacle, regardless of the environmental confounders (equivalent veiling luminance, car windshield luminance, and atmospheric luminance). In this study, an obstacle with the reflection coefficient of 20% was mounted at the entrance of the tunnel (Figure 4). Then, the car was placed near the tunnel entrance and intrinsic luminance of the road and the obstacle were measured inside the car using the luminance meter.

### Perceived contrast of obstacles

The percentage of perceived contrast of obstacle (C_perceived_) into the driver’s eyes was determined by equation 5. This percentage depends on perceived luminance of the obstacle (L_o,p_, Eq. 6) and perceived luminance of the road (L_r,p_, Eq. 7).

(Eq.5) $$C_{perceived\;}=\frac{\left[L_{O.P}-L_{r.p}\right]}{L_{r.p}}\;\times 100$$

L_O.P_=[τ_WS_. τ_atm_. L_o.intrinsic_]+[τ_WS_.L_atm_]+L_WS_+L_seq_ (Eq.6)

L_r.P_=[τ_WS_. τ_atm_. L_r.intrinsic_]+[τ_WS_.L_atm_]+L_WS_+L_seq_ (Eq.7)

## Results

### Equivalent veiling luminance

In this study, by drawing of the Holladay polar diagram on the photo of tunnel entrance (Figure 1), the percentage of environmental factors (road, rocks, meadows) was determined in each sector of the rings and the luminance of each sector of the rings was estimated based on Table 2. The luminance for each sector and for all rings (∑L_ije_) can be seen in Table 3. Thus, by equation 3, equivalent veiling luminance into the driver’s eyes was determined to be 127.5 candela per square meter. It should be noted that according to the CIE-088-2004 standard, 2 upper and 2 lower sectors of ninth ring in diagram were ignored in the calculations of luminance. These sectors, related to the dashboard and roof of car, prevent the reflection of ambient light into driver’s eyes. As a result of using the indirect method to estimate the luminance, CIE-088 standard recommendation was taken into consideration in this study. This standard expresses where the luminance is very low at the tunnel entrance rather than environment surrounding the tunnel, the luminance of tunnel entrance can be considered as zero as indicated in Table 3.

### Determining the atmospheric veiling luminance

According to guidelines, the atmospheric veiling luminance was 308 cd/m^2^, in the SSD (69.6 m) from the tunnel entrance.

### Determining the windshield veiling luminance

To measure the windshield veiling luminance, vehicle was placed in the SSD, in the summer noon. Then the luminance of a dark point on the inner wall of the tunnel was measured from both outside and inside the vehicle, and the windshield veiling luminance was determined using equation 4. Components of the windshield veiling luminance contain:

The passage of the vehicle (in the northern hemisphere): South

Luminance at the inside of vehicle: 448 cd/m^2^

Luminance at the outside of vehicle: 262 cd/m^2^

Windshield veiling luminance: 238.4 cd/m^2^

### Determining the intrinsic luminance of the road and obstacle 

At this step, an obstacle was placed at the tunnel entrance and intrinsic luminance of the road and the obstacle was measured at the entrance of tunnel and inside the car. The intrinsic luminance of the road and the obstacle include:

The passage of the vehicle (in the northern hemisphere): South

Intrinsic luminance of obstacle: 41 cd/m^2^

Intrinsic luminance of road: 17 cd/m^2^

### Determining the perceived contrast of obstacles

After determining the intrinsic luminance of the road and the obstacle, as well as the luminance of environmental confounding factors, the perceived luminance of the obstacle and the road were determined by equations 6 and 7, respectively. Moreover, perceived contrast of the obstacle at the SSD from the tunnel entrance was calculated. Given the CIE-088-2004 standard and other studies, windshield transmission coefficient (τ_ws_) and atmospheric transmission coefficient (τ_atm_) were considered as 1 and 0.8, respectively.^9^ Perceived luminance and contrast into the driver’s eyes comprises:

The passage of the vehicle: South

Luminance perceived of obstacle: 706.7 cd/m^2^

Luminance perceived of road: 687.5 cd/m^2^

Contrast perceived: 2.79%

## Discussion

The luminance of environmental confounders (equivalent veiling luminance, atmospheric veiling luminance, windshield veiling luminance) decreases the contrast of probable obstacles at the tunnel entrance. Studies conducted by Onayg and Grana et al revealed that reducing the contrast of obstacles at the tunnels entrance can raise uncertainty in making a proper decision by drivers when approaching the tunnel.^24,25^ A study by Martens et al showed that insufficient visibility leads to constant changes in the position of the vehicle and increases the driver’s fear when approaching the tunnel.^26^ Therefore, to avoid probable accidents at the entrance of tunnels, CIE 088:2004 standard recommends the minimum amount of 28% for perceived contrast of obstacle to the SSD from the tunnel entrance. In the present study, by measuring the luminance of the environmental factors, the perceived contrast of obstacle in SSD from the tunnel entrance was determined as 2.79, much less than the minimum recommended by the CIE 088:2004 (28%).

Given the environmental problems caused by the luminance of confounding factors, various studies have been conducted to improve the visibility at the tunnels entrance. A study by Onaygil et al showed that planting shrubs and trees, as well as painting concrete around the tunnel with dark colors can considerably reduce the equivalent veiling luminance into the driver’s eyes up to 57%.^27^ In the studied tunnel, not using these ways to reduce the equivalent veiling luminance of the surroundings of the tunnel was considered as one of the main reasons for low contrast of obstacle at the tunnel entrance.

The windshield veiling luminance is an important factor reducing the contrast of obstacle at the tunnel entrance. The luminance of vehicle windshield may occur directly or indirectly. In direct mode, direct striking of sunlight to the windshield and its scattering into driver’s eyes causes the glare. In indirect way, sunlight reflecting off the dashboard to windshield surface and its reflection into driver’s eyes creates the glare. The indirect luminance in the windshield is related to designing the dashboard. A study conducted by Volvo Company indicated that the plates mounted on the dashboard, especially when the sun is at its highest radiation angle [summer], cause light reflection to the windshield surface and its scattering into driver’s eyes. Consequently, this company recommended using anti-reflective coating on the dashboard surfaces to reduce the amount of glare.^17,28^ In addition to the windshield veiling luminance, a dusty, dirty, or cracked windshield can affect the luminance when driving. A study by Mefford illustrated that the presence of dirt on the inside and outside of the vehicle causes 0.000551 and 0.000516 cd/m^2^luminance per lux into the driver’s eyes, respectively.^29^ In the present study, by placing the vehicle with clean windshield in the SSD and measuring the luminance of a dark point on the tunnel wall, the luminance of windshield was calculated as 238 cd/m^2^.

Low differences between the intrinsic luminance of the road and the obstacle are also considered as main reasons to reduce the obstacles contrast at the tunnel entrance. Basically, both asymmetrical and symmetrical lighting systems are used for tunnel. In asymmetrical system, lighting is distributed against the direction of traffic, resulting in the increase of differences between the intrinsic luminance of the road and the obstacle. Symmetrical pattern is distributing the lighting in all directions to provide a uniform luminescence for the obstacle and the road surface, leading to a slight difference between the intrinsic luminance of the road and the obstacle.^14,25,30^ A study by Schreuder and Swart showed that the use of the asymmetric lighting as well as raising the contrast between the obstacle and the road surface could increase the visibility of obstacles at the tunnels entrance.^31^ At the entrance of tunnel in this study, using symmetrical lighting system had resulted in a slight difference between the luminance of the obstacle and the road. This was an effective reason for reducing the perceived contrast of obstacle at the tunnel entrance.

Taking the countermeasures such as planting trees and bushes, painting structures around the tunnel by colors with low reflection coefficient, and mounting the asymmetrical lighting system at the tunnels entrance can effectively reduce the perceived contrast of obstacles, leading to reduction of the risk of road accidents at the tunnels entrance.

One of the limitations of this study which has to be pointed out, was that the passage of vehicles in the tunnel limited the measurement of the brightness within the tunnel, so the measurement took place at a time when the traffic load was low.

The authors suggest that the effect of installation of transparent canopies at the entrance of tunnels on the improvement of contrast perception should be assessed in future studies.

## Conclusion

In conclusion, at the entrance of the studied tunnel, the contrast perception of obstacles with the reflection coefficient of 20% was lower than the minimum contrast perception (28%) recommended by CIE 088:2004. The main conclusion that can be drawn from it, is that this obstacle in the SSD from the tunnel entrance cannot be conceived by the drivers, which may lead to higher rate of road traffic crashes. In order to reach an optimum level of safety in the studied area, carrying out corrective measures and safety promotion activities at the entrance of the tunnel can be imperative.

## Ethical approval

Not applicable.

## Competing interests

The authors certify that there is no actual or potential conflict of interest in relation to this article.

## Authors’ contributions

SMJ, MHB, JS and MA conceived the study, collected the data and conducted the analysis. MK drafted the manuscript and wrote the manuscript. SFD translated, interpreted data, and reviewed the manuscript. AM and RH oversaw the whole study process, designed the study, analyzed and interpreted data, and critically reviewed the manuscript. All authors have reviewed and provided intellectual feedback on the manuscript. All authors have read and approved the final version of the manuscript and agree with the order of presentation of the authors.

## Supplementary Materials


Supplementary file 1 contains Figures S1-S2.

## Acknowledgments

Authors would like to thank the General Directorate of Roads and Urban Development of Ilam province for providing organizational information.

**Table 1:** Rings angle of the Holladay polar diagram

**Ring (In to out)**	**Central**	**1**	**2**	**3**	**4**	**5**	**6**	**7**	**8**	**9**
**Angle (°)**	2	3	4	5.8	8	11.6	16.6	24	36	56.8

**Table 2:** Estimation of the luminance in each sector of the polar diagram based on CIE-088-2004 standard

**Driving directions (in the northern hemisphere)**	**The luminance of the road (kcd/m** ^ 2 ^ **)**	**Luminance of tunnel entrance (kcd/m** ^ 2 ^ **)**	**The luminance of the surrounding environment of the tunnel (kcd/m** ^ 2 ^ **)**		
			**Rock**	**Building**	**Meadows**
South	5	0	1	4	2

**Table 3:** The luminance for each sector of the rings of the Holladay diagram in the south line of the tunnel entrance

**Sectors**	**Rings**								
	**1**	**2**	**3**	**4**	**5**	**6**	**7**	**8**	**9**
1	5	5	5	5	5	5	5	5	Not calculated
2	0	5	5	5	5	5	5	5	5
3	0	0	0	2.9	4.4	4.7	4.3	4.7	4.7
4	0	0	0	0.2	3.7	2.8	1	1	1
5	0	0	0	0	0.95	1	1	1	1.8
6	0	0	0	0.05	1	1	1	1.5	Not calculated
7	0	0	0	0.1	1	1	1	1.6	Not calculated
8	0	0	0	0	0.55	1	1	1.1	2
9	0	0	0	0	3.5	3.6	1.15	1	1.02
10	0	0	0	2.5	4.5	4.8	4.8	4.8	4.8
11	0	2.5	5	5	5	5	5	5	5
12	5	5	5	5	5	5	5	5	Not calculated
Luminance of each ring	10	17.5	20	25.75	39.6	39.9	35.25	36.7	25.32
Luminance of all rings					∑L_ije_ = 250.02 Kcd/m^2^
