# Supplementary material for: Assessment of contrast perception of obstacles in a tunnel entrance
Source: Health Promot Perspect. 2018 Oct 27;8(4):268–74. doi: 10.15171/hpp.2018.38 (PMC6249494; doi:10.15171/hpp.2018.38)
Supplement: Supplementary file 1 — contains Figures S1-S2. [file hpp-8-268-s001.pdf]

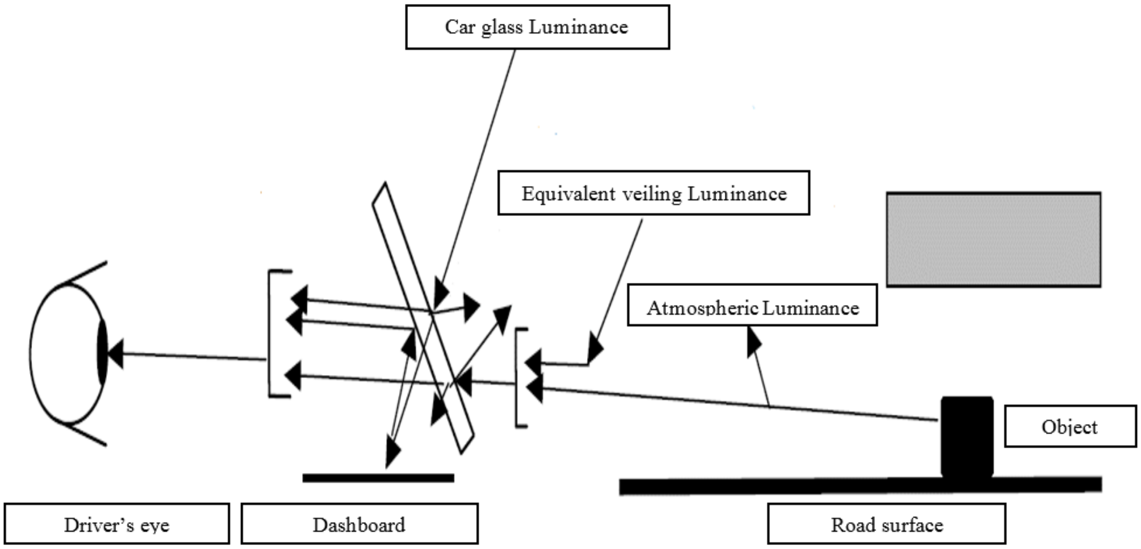

Figure S1. Factors affecting the disability glare at the entrance of tunnels.[9]

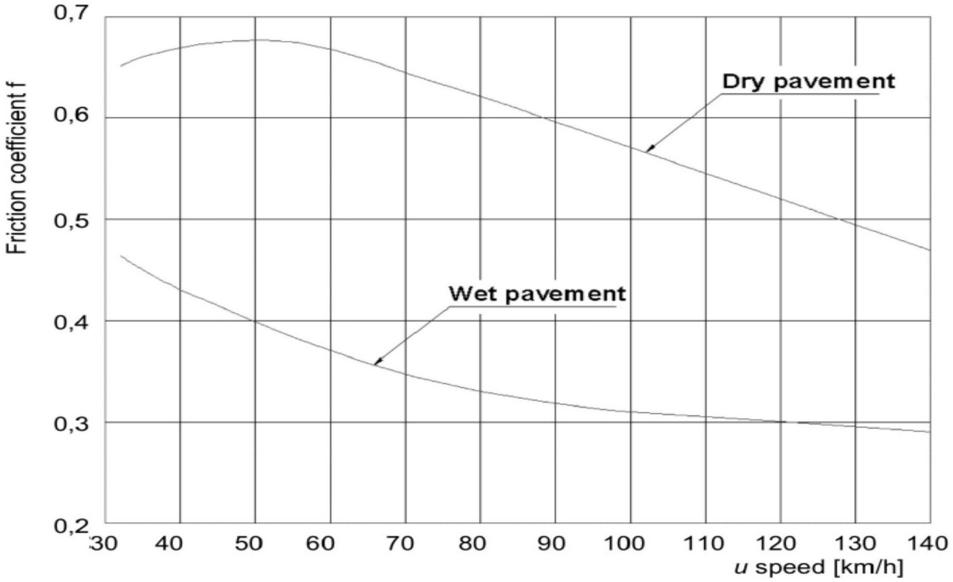

Figure S2. Determination of the coefficient of friction between the tire and the road.[9]
